# Rising Star Early-Career Faculty Award Lecture

**DOI:** 10.1093/geroni/igaa057.3211

**Published:** 2020-12-16

**Authors:** Cynthia Hancock

## Abstract

The Rising Star Early-Career Faculty Award lecture will feature an address by2020 recipient Laurinda Reynolds, MA. The Rising Star Early-Career Faculty Award acknowledges new faculty whose teaching and leadership stand out as influential and innovative.

